# Simpson grade 3 resection does not improve clinical outcome in neglected thoracic psammomatous spinal meningioma? A case report

**DOI:** 10.1016/j.ijscr.2019.07.033

**Published:** 2019-07-22

**Authors:** Azharuddin Azharuddin, Muhammad Bayu Zohari Hutagalung, Reno Keumalazia Kamarlis

**Affiliations:** a Department of Orthopaedic and Traumatology, Faculty of Medicine, Syiah Kuala University/Dr. Zainoel Abidin Hospital, Banda Aceh, Indonesia; b Department of Pathology Anatomy, Faculty of Medicine, Syiah Kuala University/Dr. Zainoel Abidin Hospital, Banda Aceh, Indonesia

**Keywords:** Intramedullary, Psammomatous, Spinal meningioma, Neglected, Resection

## Abstract

**Introduction:**

Thoracic spinal psammomatous meningioma is a rare subtype of meningioma. Surgery is the definitive treatment for symptomatic spinal meningiomas and offers a substantial possibility for complete resection and cure.

**Presentation of case:**

A 42-year-old female complaining of back pain for one year and progressive weakness and numbness of both lower limbs with urinary incontinence and constipation for two months. Magnetic resonance imaging showed an intramedullary spinal mass at D3-D4 level. The patient underwent laminectomy at D3-D5 level, revealing dural bulge. A midline durotomy performed. Intraoperative findings showed firmed, greyish to white and moderately vascular mass. A clear margin was identifiable between cord and tumor. Simpson grade 3 resection was performed. Pedicle screw and rod was placed for posterior stabilization. Histopathological examination demonstrated a psammomatous spinal meningioma. Despite the pain and the sensory sensation was improved, the motoric and autonomic function still showed no significant improvement in the early postoperative period.

**Discussion:**

The preferred approach or procedure must be tailored case by case based on preoperative surgical grading of the tumor and its associated factors. Meanwhile, the prognosis for recovery depends mainly on two factors: the severity of the neurological deficit and the duration of the deficit before decompression.

**Conclusion:**

Surgical resection of the tumor may relieve the spinal cord compression in a neglected case. Even though the neurologic function after surgery rarely returns to the functional stage due to chronic neural tissue damage.

## Highlights

• Thoracic spinal psammomatous meningioma is a rare subtype of meningioma often present with pain, sensory-motor deficit, and sphincter disturbance.
• Neglected spinal cord compression can result in a permanent neurological deficit.
• Surgical resection of the tumor relieved the spinal cord in a neglected case.
• The severe preoperative neurological deficit as a predictor of a poor prognosis.
• The neurological function after surgery hardly returns to the functional stage in a neglected case.

## 1 Introduction

Spinal meningioma accounts for 25%–46% of all intraspinal tumors, 85% of which are intradural, 7% with extradural extension, and 8% entirely extradural [1,2]. They are common in the middle age group, and most of them are benign, most of them being nerve sheath tumors and meningioma. The early diagnosis and surgical removal and relieving pressure on the cord, along with intensive rehabilitation, give excellent results with the best outcome [3,4]. We report a rare case of intramedullary psammomatous meningioma of the thoracic spine in a 42-year-old female. Also, we discuss the pathogenesis of such tumors and the potential challenges in differential diagnosis and review the associated literature focusing on the treatment and outcome. The following case has been reported in line with SCARE criteria [5].

## 2 Presentation of case

**Fig. 1:**
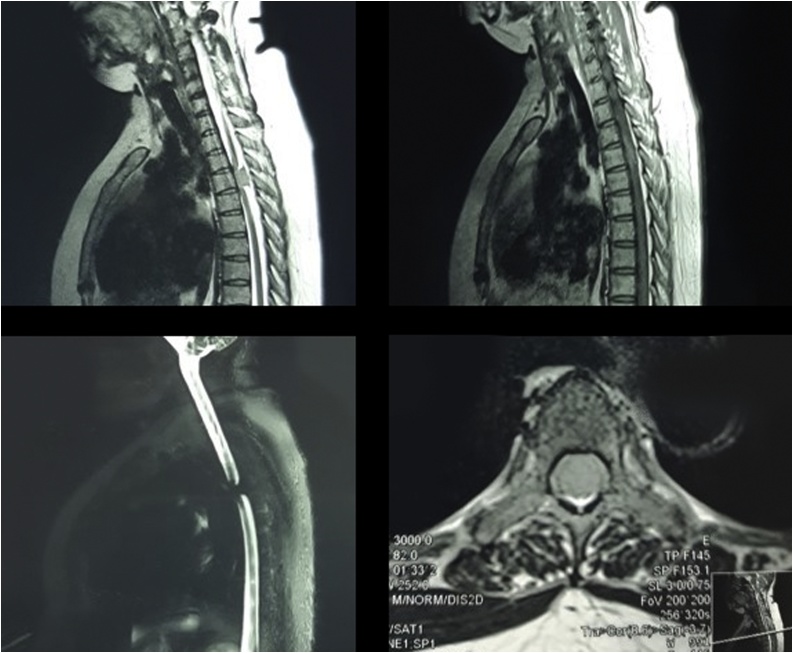
Magnetic Resonance Imaging revealed hypointense lesion extending from midbody of D3 to midbody of D4 vertebra.

A 42-year-old female presented with chief complaints of gradually progressing weakness and diminished sensation in both the lower limbs of 1-year duration. For the last two months, she started experiencing constipation and urinary incontinence. These complaints preceded by pain in the dorsal spine. Her lower limb weakness progressed, and she became bedridden. There was no history of fever, trauma, or any chronic illness. On physical examination, spastic paraplegia and loss of all sensory modalities below the D5 dermatome were found. The neurological condition showed severe deficit and pain based on Modified McCormick Functional Scheme (mMFS) and Sensory Pain Scale (SPS). The Magnetic Resonance Imaging (MRI) of the dorsal spine revealed an intramedullary spinal lesion extending from midbody of D3 to midbody of D4 vertebra, which was hypointense on T3 and T4 (Fig. 1).

**Fig. 2:**
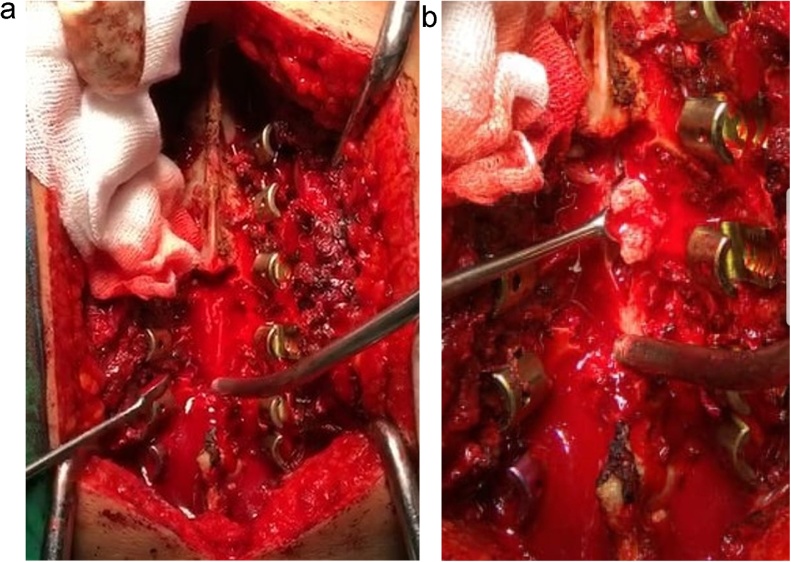
(A) Intraoperative image showing midline durotomy at the T3–T5 level; (B) Intraoperative findings showed soft to firm, greyish white and moderately vascular mass as an intradural extramedullary tumor.

**Fig. 3:**
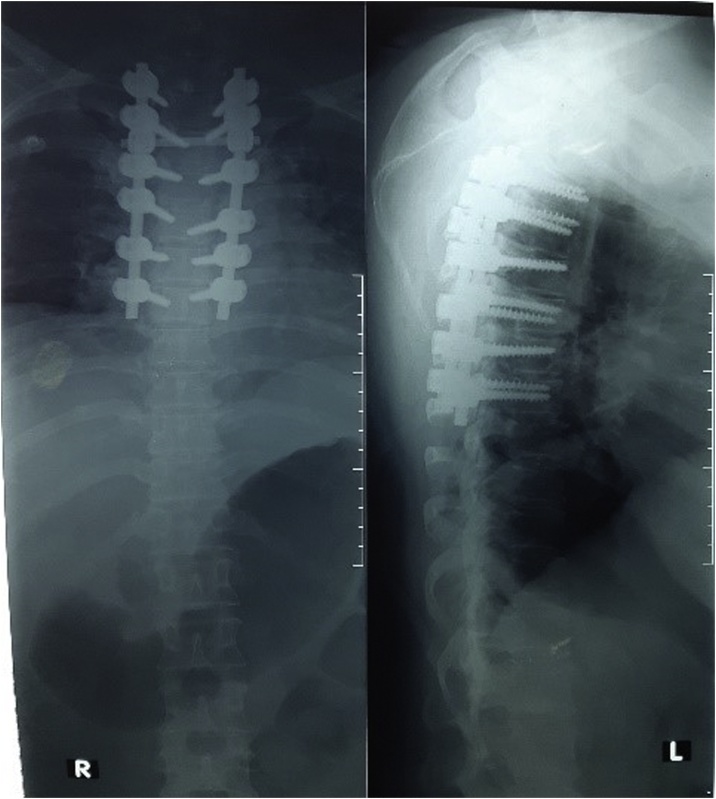
The postoperative radiograph showed pedicle screw and rod placement for posterior stabilization.

**Fig. 4:**
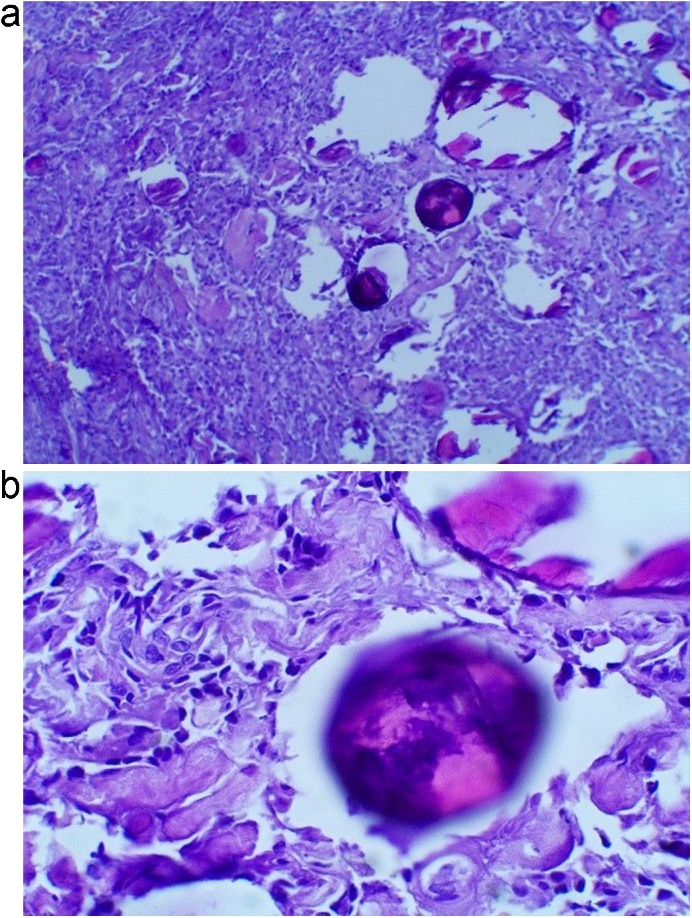
(A) Photomicrographs of tumor showing multiple calcified psammoma bodies; (B) magnification of psammoma bodies (Hematoxylin-eosin stain; left and right magnification; 100× and 400×).

The patient underwent D2–D5 laminectomy revealing dural bulge. A midline durotomy followed by a midline myelotomy between the dorsal columns. The tumor was encountered after the columns were split. Intraoperative findings revealed a soft to firm, greyish white and moderately vascular — (Fig. 2). Simpson grade 3 resection was performed. Pedicle screw and rod was placed for posterior stabilization (Fig. 3). Histopathological examination reported features suggestive of psammomatous meningioma (Fig. 4). Even though patient pain and lower limbs sensation improved in the postoperative period, the motoric and autonomic function still showed no improvement at all. The patient was discharged after a week later and planned for medical rehabilitation in outpatient care service.

## 3 Discussion

Spinal meningioma arises from arachnoid villi related to emerging nerve roots and are located intradural in most cases [6]. The definitive diagnosis delay from the onset of symptoms was commonly found in this case. A study by Pesna et al. found a median delay in diagnosis of 24 months (range three days to 24 years) amongst 57 patients, referred to them between 1978 and 1988 [7]. The majority of patients often present with pain, sensory-motor deficit, and sphincter disturbances. Generally, the back pain symptom precedes the onset of motoric weakness and sensory changes. While the sphincter dysfunction is always a late finding. The clinical features between an extradural and intradural meningioma showed no significant difference [8].

The imaging study using MRI is the best imaging option for diagnosing spinal meningiomas. It delineates the level of the tumor and its relations to the cord, which is useful in planning surgery [1,9]. The appearance of iso- or hypointensity mass on T2 MRI sequences in contrary with the T2 hyperintensity mass of most epidural tumors, except for lymphomas that can be hypointense in over 50% of cases [4,9].

Surgery is the definitive treatment for symptomatic spinal meningiomas and offers a substantial possibility for complete resection and cure. Neglected and prolonged spinal cord compression due to a spinal meningioma can result in a permanent neurological deficit even after surgery. Posterior laminectomies indicated for the mass that located posteriorly. If the tumor located anteriorly, the tumor removal can be counter through extended lateral laminectomy towards the articular process, which gives excellent exposure with minimal displacement of the spinal cord or an anterior approach via posterolateral thoracotomy [7]. With total excision of the tumor, recurrence-free survival rates of 93%, 80%, and 68% at 5, 10, and 15 years can be anticipated [10]. However, relatively poor outcomes are associated with subtotal resection, the presence of an extradural component, young age, multiple lesions, calciﬁcation, ossiﬁcation, and anterior location of the lesion [11]. The present case had an intradural component, single foci of intratumoral, and no sign of dural calciﬁcation and ossiﬁcation. Simpson grade III resection performed in this case owing that the dura is normal and uninvolved with the tumor.

Our patient showed clinical improvement in back pain and sensory function. Meanwhile, the motoric function showed no significant improvement in the early follow-up. The prognosis for recovery depends mainly on two factors: the severity of the neurological deficit and the duration of the deficit before decompression. Spinal cord compression damages neural tissue both by mechanical and vascular mechanisms, especially in the chronic and neglected spinal meningioma as presented in this case. Since then, large series have confirmed the usually favorable outcome following removal of these tumors but have also emphasized the importance of the preoperative neurological deficit as a predictor of a poor outcome [2,12].

A tendency of psammomatous meningiomas, the histologic subtype of the present case, toward pial invasion is well known, which further challenges surgical resection. Although overall prognosis in meningioma depends in large part on the entirety of resection, the extradural ossiﬁed meningioma should be approached judiciously to avoid postoperative neurologic deﬁcits. Regardless of the extent of dural removal, a prime goal throughout the surgery should be the maintenance of minimal or no retraction of the spinal cord, as this is likely to be associated with favorable immediate postoperative and long-term outcomes as in the present case [11]. The current perspective noted that adjuvant radiation therapy Another standpoint is better to reserve for recurrent, difficult to reach, high-grade cases than after first surgery incorporating the risk of irradiating a functional spinal cord with no proven benefit, especially the lesion that was located in the anterior and difficult to reach without spinal cord radiation exposure [7]. The preferred approach or procedure must be tailored case by case based on preoperative surgical grading of the tumor and its associated factors.

## 4 Conclusion

Neglected spinal meningioma associated with severe and chronic neurologic deficit. The mass effect of the tumor, causing chronic spinal cord compression leading to permanent neural tissue damage due to both mechanical and vascular mechanism. Despite, surgical resection of the tumor relieved the spinal cord compression in neglected case. The neurologic function after surgery rarely returns to the functional stage.

## Sources of funding

None.

## Ethical approval

This study has been approved by the local ethic Committee (Faculty of Medicine, Syiah Kuala University, Banda Aceh).

## Consent

The patient consent regarding this study was obtained.

## Author contribution

All author has been involved in all stage of study concept and writing the paper.

## Registration of research studies

None.

## Guarantor

Muhammad Bayu Zohari Hutagalung, MD.

## Provenance

Not commissioned

## Declaration of Competing Interest

None.
